# Disk solid-phase extraction of multi-class pharmaceutical residues in tap water and hospital wastewater, prior to ultra-performance liquid chromatographic-tandem mass spectrometry (UPLC-MS/MS) analyses†

**DOI:** 10.1039/c8ra06885b

**Published:** 2018-12-03

**Authors:** Husam I. S. Kafeenah, Rozita Osman, N. K. A. Bakar

**Affiliations:** Department of Chemistry, Faculty of Science, University of Malaya 50603 Kuala Lumpur Malaysia kartini@um.edu.my; Faculty of Applied Sciences, Universiti Teknologi MARA 40450 Shah Alam Selangor Malaysia

## Abstract

In this work, a new clean-up and pre-concentration method based on disk solid-phase extraction (SPE) was developed to determine multi-class pharmaceutical residues covering a wide range of polarities (log *K*_ow_ values from −0.5 to 5.1) in water systems, prior to ultra-performance liquid chromatographic-tandem mass spectrometry (UPLC-MS/MS) analyses. Electrospray ionisation in positive and negative modes was used for the simultaneous determination of both acidic and basic pharmaceuticals. The performances of disk SPE and cartridge SPE were compared. The targeted pharmaceutical compounds list included bronchodilators, antidiabetic drugs, antihypertensive drugs, a lipid-lowering agent, analgesics, and anti-inflammatory drugs. Based on our results, the disk SPE demonstrated a higher sensitivity and recovery value and less analysis time as compared to the cartridge SPE method. The limits of detection (LOD) for the new method ranged from 0.02–3.2 ng L^−1^, 0.02–3.1 ng L^−1^ and 0.02–4.7 ng L^−1^ for tap, effluent and influent wastewater, respectively. The method's absolute recovery values ranged from 70% to 122% for tap water, 62% to 121% for effluent wastewater and 62% to 121% for influent wastewater, except for metformin in which the absolute recovery value was approximately 48% for all samples. Intra-day precision for tap water, effluent and influent wastewater ranged from 3–12%, 4–9% and 2–8%, respectively. The method developed was applied for the determination of targeted pharmaceuticals in tap, effluent, and influent wastewater from one hospital treatment plant in Malaysia. The results revealed that the highest concentrations of certain pharmaceuticals were up to 49 424 ng L^−1^ (acetaminophen) and 1763 ng L^−1^ (caffeine) in the influent and effluent wastewater, respectively. The results also showed a variation in the treatment efficiencies for the hospital treatment plant from one compound to another. Nevertheless, the removal efficiencies ranged from 0–99%.

## Abstract 2

A new sensitive analytical method was developed for pharmaceuticals with a very wide range of polarities, and log *K*_ow_ ranging from −0.5 to 5.12.

## Graphical abstract

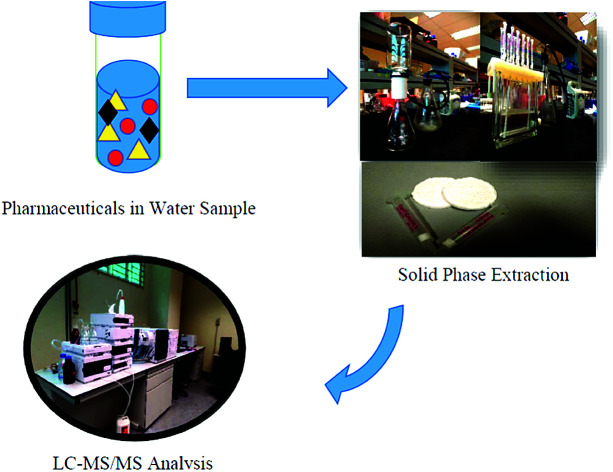

## 1 Introduction

With the worldwide increase in pharmaceutical consumption, tracing pharmaceutical pollution in water has recently become a concern. Good analytical methods are the key to tracing and understanding the fate of pharmaceutical pollution in water systems. Therefore, sample preparation is a crucial step in trace analysis for the clean-up and pre-concentration of samples with various concentrations and properties of analytes in a short time, in order to eliminate the matrix interferences in the instrumental analysis and obtain a sensitive and robust analytical method.^1–4^ Several types of cleaning and pre-concentration techniques have been used to clean-up pharmaceuticals in water; *e.g.*, liquid–liquid extraction (LLE) and solid-phase extraction (SPE) methods.^5–7^ Over the past decade, SPE has gradually replaced LLE methods as the favoured and most popular technique for extracting pharmaceuticals from water *prior* to quantitative analysis, due to the simplicity, robustness and the small amount of solvent used.^1,8–12^

Several SPE formats have been developed in order to suit the different types of samples starting from simple packed disposable syringes to cartridges, disks, SPE pipette tips, 96-well, and 384-well microplates.^13^ The SPE cartridge is the most popular SPE format in water analyses.^14–16^ Most of the previous studies used cartridge formats in SPE clean-up, despite a lot of drawbacks when applied in real samples such as the high potential for blockage and the low flow rate (due to the small cross-sectional area in the packing material) that lead to the increase in the analysis time and difficulty handling large volumes of sample.^7,17–19^ Moreover, the capacity to retain the analytes is low as a result of the large particle size of the packing material in the cartridge. In order to overcome these drawbacks, SPE disk formats have been developed to enhance the extraction performance. SPE disks are made of rigid glass or PTFE fibre material embedded with bonded silica or polymer and the sorbent material. The sorbent particles embedded in the disks are smaller than those in the cartridges (8 μm diameter rather than 40 μm to 60 μm). Smaller disk particle size allows for more interaction between the analytes and the sorbent material. Therefore, the SPE disk is more prone to have efficient trapping of analytes from the water sample, which leads to enhancing the recovery of the methods.^18^ Furthermore, the short sample path and small particle size in the disk allow the use of greater volume with high flow rates of samples (even the unfiltered samples) and reduce the eluting material amount, which means improving the limit of detection and quantification without increasing the analysis time and at the same time reducing the risk of plugging.^17^ Several studies have confirmed good recoveries and performance in determining herbicides and pesticides in water by using SPE disks.^5,6,20–23^

High-performance liquid chromatography-mass spectrometry is one of the most popular instruments in pharmaceutical analysis.^24^ Studies have been conducted in order to improve the liquid chromatography-mass spectrometry (LC-MS) technology and overcome the limitations.^25,26^ One of these limitations is the difficulty in running acidic and basic compounds simultaneously, due to the requirements for different ionisation modes for different compounds; acidic compounds require a negative ionisation mode and basic compounds operate in positive ionisation mode. With an increase in switching speed between the negative and positive modes in ESI-LC-MS, a new method that simultaneously employs both the negative and positive electrospray ionisations (ESI) was developed to obtain the maximum amount of information in a short time and with good sensitivity for a wide variety of compounds with different physicochemical properties.^27,28^

Most of the previous research in pharmaceutical analysis targeted compounds belonging to one or two pharmaceutical classes with similar properties, while only a few studies targeted multi-class pharmaceutical compounds in wastewater. The detection of multi-class pharmaceuticals with different physicochemical properties could reduce the efficiency of the extraction process, thereby reducing the sensitivity of the detection of such compounds as a consequence of different polarities being retained differently on the adsorbent when preparing the sample. The more chemically different the analytes are, the more difficult it is to develop the analytical method with acceptable recovery and sensitivity for their detection in a matrix. Most of the previous research attempted to enhance the extraction efficiency of multi-class pharmaceuticals by testing different adsorbing materials. To the best of the authors' knowledge, none of the previous studies have tried to improve the extraction efficiency of multi-class pharmaceutical residues in water by testing different formats of SPE (cartridges and disk).^29–32^ No previous study has been carried out for water analysis with this level of sensitivity involving groups of pharmaceuticals with variations in properties such as p*K*_a_ ranging from 3.7 (perindopril) to 12.4 (metformin) and a wide range of polarities, with log *K*_ow_ ranging from −0.5 to 5.12. This is also the first SPE method used for the detection of metformin in water samples with this level of sensitivity.

In this work, the performances of the disk SPE and cartridge SPE were compared to determine the more efficient SPE format for the clean-up and pre-concentration of 10 multi-class pharmaceuticals with a wide range of polarities from water samples, and thus a sensitive analytical method for the determination of multi-class pharmaceuticals was developed. The properties and classes of the pharmaceutical compounds, including bronchodilators, antidiabetic drugs, antihypertensive drugs, lipid-lowering agents, analgesics and anti-inflammatories are given in Table 1 (ref. 33) and Fig. S1 (ESI†). Ultra-high performance liquid chromatography-tandem mass spectrometry (UPLC-MS/MS) utilising triple quadrupole (QQQ) mass spectrometry was employed as a detector. Both positive and negative ionisation modes in electrospray ionisation (ESI) were used simultaneously. The developed method was used investigate the occurrence of these pharmaceuticals in tap water, effluent, and influent wastewater samples, and the efficiency of the removal process in a hospital wastewater treatment plant was assessed.

**Table 1:** Properties of pharmaceuticals and their applications^a^

Compound name	Application origin	MW	log *K*_ow_	p*K*_a_	Molecular formula	Water solubility (at 25 °C) mg L^−1^
Acetaminophen	Analgesics/anti-inflammatories	151.1	0.4	9.8	C_8_H_9_NO_2_	1.4 × 10^4^
Caffeine	Stimulants/caffeine metabolites	194.1	−0.1	10.4	C_8_H_10_N_4_O_2_	2.2 × 10^4^
Diclofenac	Analgesics/anti-inflammatories	296.1	4.5	4.2	C_14_H_11_Cl_2_O_2_	2.4
Ibuprofen	Analgesics/anti-inflammatories	206.2	3.9	4.9	C_13_H_18_O_2_	21.0
Mefenamic acid	Analgesic and anti-inflammatories	241.2	5.1	4.2, −1.6	C_15_H_15_NO_2_	20.0
Metformin	Anti-diabetic	129.1	−0.5	12.4	C_4_H_11_N_5_	1.1 × 10^6^
Nifedipine	Antihypertensive	346.3	2.2	5.3, 3.9	C_17_H_18_N_2_O_6_	0.02
Perindopril	Antihypertensive	368.4	2.6	3.7, 5.4	C_19_H_32_N_2_O_5_	1.2
Salbutamol	To treat asthma, agonists bronchodilator	239.3	0.4	10.3	C_13_H_21_NO_3_	1.4 × 10^4^
Simvastatin	Lipid-lowering agent	418.5	4.6	14.9, −2.8	C_25_H_38_O_5_	0.01

a Source (The DrugBank Database).^33^

## 2 Materials and method

### 2.1 Chemicals and materials

Methanol (MEOH), acetonitrile (ACN), acetic acid (AcOH), and ammonium acetate (AmAc) were supplied by Merck (Darmstadt, Germany) and all the mobile phase solvents and reagents were high-performance liquid chromatographic (HPLC)-grade with ≥99% purity. Ultra-pure water was prepared from a Milli-Q water purification system (MA, USA). Standard pharmaceuticals (metformin, ibuprofen, mefenamic acid, simvastatin, caffeine, diclofenac, perindopril, and nifedipine) were purchased from Sigma-Aldrich (Schnelldorf, Germany). The acetaminophen reference standard material was purchased from Sigma-Aldrich (St. Louis, MO, USA). The purity of all standards used in this study was ≥98%. A 1000 mg L^−1^ stock standard solution was prepared for each pharmaceutical by dissolving an appropriate amount of each analytical standard of pharmaceuticals in methanol. Working solutions (5 mg L^−1^) were prepared from the 1000 mg L^−1^ stock solutions by adding 50 μL of each stock solution to a 10 mL volumetric flask and then methanol was used to fill it up to the mark. A series of standard solutions for the calibration was conducted using the working solution and diluting with water: methanol (2 : 1 v/v) at pH 10. The mixture was adjusted to pH 10 using ammonium hydroxide, 1 M.

### 2.2 Sample collection and preparation

Three types of water samples were collected: tap water, effluent and influent wastewater. Tap water was collected from a Research Laboratory at the University of Malaya, while the wastewater was collected from a wastewater treatment plant at Sungai Buloh Hospital, Malaysia. All water samples were collected using a homemade glass sampler. Then, the samples were kept in clean 1 L amber glass bottles. The bottles were stored under ice in an icebox at 0 °C while the samples were transported to the laboratory. Water samples were preserved by adding 1 g of sodium azide per liter of sample to prevent microbial degradation, and 50 mg of ascorbic acid per liter of sample to quench any residual oxidation by chlorine, chloramine and ozone.

### 2.3 Solid-phase extraction (SPE)

#### 2.3.1 Disk extraction

Atlantic HLB (hydrophilic–lipophilic balance) disposable disks from Horizon Technology (Northwestern, USA) were used for SPE. The volumes of samples were 200 mL, 500 mL and 1000 mL for sewage influent, effluent and tap water, respectively. All the samples were filtered through a GF 6 glass membrane filter (Schleicher & Schuell). Then, the pH of the water samples was adjusted to 7.0 with 1 M hydrochloric acid (HCl) and 1 M sodium hydroxide (NaOH). Finally, the disk adsorbent material was pre-conditioned with 5 mL of methanol followed by 5 mL of ultrapure water at pH 7.0.

The disks were placed on 47 mm disk holders and the samples were introduced under vacuum at flow rates of 20, 50 and 100 mL min^−1^ for sewage influent, sewage effluent and tap water, respectively. After sample loading, the disk was washed with 5 mL of 5% methanol in ultrapure water at pH 7. The disk was then dried for about 30 min and subsequently, the analytes were eluted twice with 1 mL of a mixture of ACN : MEOH (1 : 1 v/v) with 2% formic acid, then twice with 1.5 mL of the mixture of ACN : MEOH (1 : 1 v/v) with 2% ammonium hydroxide. The extracts were evaporated to near dryness under a vacuum at 50 °C, then reconstituted in 0.5 mL of methanol and diluted to 2 mL using water: methanol (2 : 1 v/v) at pH 10 (Fig. S12, ESI†). The extracts were stored at −18 °C until analysis.

To determine the best sample pH and the optimum eluent material and additive material, different pH samples and several eluent materials with different additive materials at various concentrations were tested for all the pharmaceuticals together using the described method above on spiked ultrapure water by varying one parameter at a time. The sample pH values were adjusted using 1 M HCl and 1 M NaOH solution, while ammonium hydroxide and formic acid were used as additive materials.

#### 2.3.2 Cartridge extraction

HLB cartridges (3 mL, 60 mg) were conditioned with 4 mL of methanol followed by 5 mL of water at pH 7, then the samples were loaded using a large volume sampler at flow rates of 4, 10 and 20 mL min^−1^ for sewage influent, sewage effluent and tap water, respectively. After all the samples were loaded, the cartridges were rinsed with 3 mL of 5% methanol in ultrapure water at pH 7, dried and eluted twice with 1 mL of the mixture of ACN : MEOH (1 : 1 v/v) with 2% formic acid, then twice with 1.5 mL of the mixture of ACN : MEOH (1 : 1 v/v) with 2% ammonium hydroxide. The extracts were evaporated to dryness, then reconstituted in 0.5 mL of methanol and diluted to 2 mL using (2 : 1 v/v) water : methanol at pH 10. All the method validation tests for the disk method were applied for the cartridge in order to compare the performance of both SPE methods.

### 2.4 UPLC-ESI-MS/MS analysis

Agilent 1290 UPLC was used for the liquid chromatographic (LC) analysis (Agilent Technologies, Germany). A reverse-phase Accucore Polar Premium LC column (100 mm × 2.1 mm, particle size 2.6 μm, Loughborough, UK) was utilized for the chromatographic separation. The injection volume was set at 3 μL and the column temperature was 35 °C. For the mobile phase, methanol was used as the organic eluent and AmAc, 0.0012 M/AcOH in HPLC water at pH 4.6 was used as the aqueous eluent at flow rates of 0.2–0.25 mL min^−1^ (Fig. S13 (ESI†)).

A gradient elution programme was developed. The composition of the mobile phase started with 10% methanol at a flow rate of 0.2 mL min^−1^ for 1 min. The methanol was elevated from 10 to 80% at a flow rate of 0.2 mL min^−1^ over the following 5 min, then the methanol was increased to 100% at a flow rate of 0.2 mL min^−1^ over the next 4 min; this was held for 0.5 min. Finally, the methanol ended with 10% in 1.5 min at a flow rate of 0.25 mL min^−1^. The system was allowed to equilibrate for 4 min before each injection.

An Agilent 6490 triple-quadrupole mass spectrometer (Agilent Technologies, Singapore) with Agilent Jet Stream system AJS ESI electrospray ionisation was used to detect the analytes. Both positive and negative ionisation modes were operated simultaneously. Capillary voltage was 2 kV and the nebuliser pressure was 45 psi for both modes. Nitrogen gas was used for both dissolution and nebulising gas at a flow rate of 14 L min^−1^ and temperature of 225 °C, with a dwell time at 0.2 s Table 2.^34^ In addition, the MassHunter software was used for instrument control, peak detection, and integration. To increase sensitivity, selectivity, and data acquisition, multiple reaction monitoring modes (MRM) were used.

**Table 2:** Mass spectrometry parameters for each pharmaceutical^a^

Compound Name	Polarity	*R*_t_	Precursor ion	Product ion 1	Fragmentation pattern^34^	CE	Product ion 2	Fragmentation pattern^34^	CE
Acetaminophen	Positive	3.9	152.0	109.9	[M–CH_3_]^+^	13	65.1	[M–CH_2_CO + H]^+^	33
Caffeine	Positive	4.4	195.0	138.1	[M–N_2_C_2_H_4_]^+^	21	42.1	—	40
Diclofenac	Positive	8.5	296.0	214.0	[M–ClCO_2_]^+^	33	215.0	—	17
Ibuprofen	Negative	8.1	205.1	159.0	[M–H–CO_2_]^+^	2	161.0	—	2
Mefenamic acid	Positive	9.6	242.2	224.2	[M–H_2_O]^+^	13	209.1	[M–H_2_O–CH_3_]^+^	29
Metformin	Positive	1.4	130.1	59.9	[M–C_3_N_2_H_8_]^+^	13	71.1	[M–CN_3_H_4_]^+^	21
Nifedipine	Positive	7.3	347.1	315.1	—	1	254.2	—	13
Perindopril	Positive	6.4	369.0	172.1	[M–C_10_O_3_NH_18_] ^+^	21	98.0	—	40
Salbutamol	Positive	3.5	240.3	148.2	[M + H–(CH_3_)_2_C–CH_2_− (H_2_O)_2_]^+^	13	222.2	—	5
Simvastatin	Positive	8.9	419.2	199.3	[M–(CH_3_)_2_**–**COH_2_]^+^	1	285.2	[M**–**H_2_O**–**C_6_O_2_H_12_]^+^	9

a CE: collision energy, *R*_t_: retention time.

### 2.5 Quantification and method validation

Peak area was used for quantification purposes. Nine points calibration curve with concentration levels in the range of 0.1–12 000 ng L^−1^ (except for acetaminophen the range of 10–60 000 ng L^−1^) was constructed. Each point was obtained by injecting the extract of the ultrapure water with a mixture of the pharmaceuticals into LC-MS/MS. The limits of detection (LOD) and limits of quantification (LOQ) were evaluated by measuring the concentrations of the SPE spiked samples, where the signal-to-noise ratios were 3 and 10, respectively. The samples were spiked at low concentration (0.02–10 ng L^−1^). Recoveries for all matrixes were determined by analyzing three spiked replicates of each sample matrix (at high and low concentration levels) by the described SPE method and LC-MS/MS method. Then, the concentrations of the spiked samples (the concentration of non-spiked water was subtracted) were compared to the concentrations of non-extracted standard solutions. The spiking levels were 50, 1000 and 500 ng L^−1^ for the low concentration levels and 500, 10 000 and 5000 ng L^−1^ for the high concentration levels of tap water, wastewater influent and effluent samples, respectively.

Intra-day precision was calculated as the relative standard deviation for five spiked replicates extracted and analysed on the same day, while for inter-day precision, one spiked sample was extracted and analysed on five different days. Intra-day and inter-day precision spiked samples were spiked at 3 different concentration levels (5, 50 and 500 ng L^−1^ for tap water and 100, 1000 and 9000 ng L^−1^ for influent wastewater and 50, 500 and 5000 ng L^−1^ for effluent wastewater). Signal suppression was evaluated by comparing the change in the peak intensity between the sample matrix *versus* ultrapure water, then calculated by the following equation:

1 Signal suppression (%) = (1 − (*I*_s_ − *I*_o_)/*I*_MQ_) × 100

where *I*_s_ was the compound peak intensity in the blank matrix extract spiked after extraction with a mixture of pharmaceuticals (100 ng L^−1^); *I*_o_ was the compound peak intensity in the non-spiked blank matrix extract, and *I*_MQ_ was the compound peak intensity in ultrapure water extract spiked after extraction with the same mixture.

## 3 Results and discussion

### 3.1 Sample collection and pharmaceutical selection

Influent and effluent wastewaters were collected from Sungai Buloh Hospital's wastewater treatment plant. The hospital is located in Selangor, Malaysia. The hospital serves the districts of Gombak, Petaling and Kuala Selangor with a combined population of more than 2.80 million. The hospital water treatment plant provides primary and secondary treatments, which include mechanical treatment and oxidation ponds. The treatment process is based on using the activated sludge bacteria to “eat” small organic carbon molecules. The wastewater samples were collected during three sampling campaigns: one in the dry season (Oct 2016) and two in the rainy season (May and Aug 2017). The 10 pharmaceutical compounds in this study were selected out of the top 40 pharmaceuticals prescribed in Malaysia, according to the National Medicines Use Survey 2015.^35^ The selected pharmaceuticals belong to six different therapeutic categories to ensure the variation in polarity.

### 3.2 Solid phase extraction

#### 3.2.1 Selecting the adsorbent material

HLB polymeric sorbent material was selected in this study because it is the most frequently used adsorbent material in the multi-analysis of pharmaceuticals in water.^18,27,30^ The advantage of using HLB as compared to other sorbent materials is the lipophilic divinylbenzene units and the hydrophilic *N*-vinylpyrrolidone units, which make it suitable for the pharmaceuticals with different chemical properties and polarities.^9^

#### 3.2.2 pH selection of sample

**Fig. 1:**
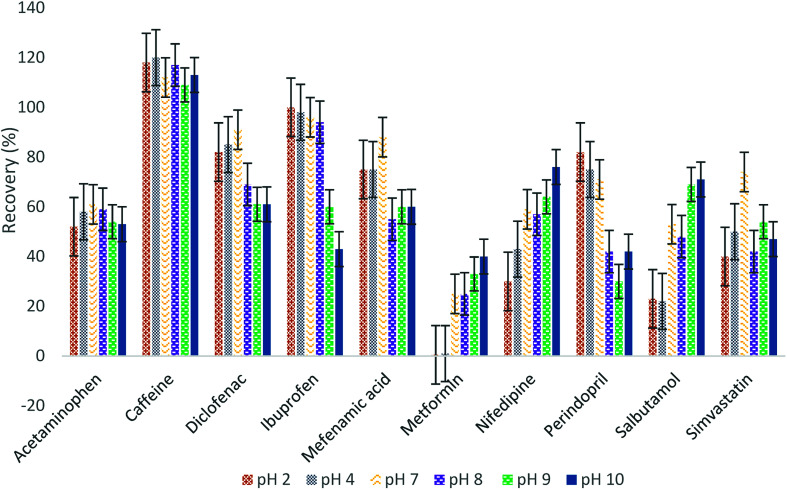
Effect of sample pH on the recovery of pharmaceuticals.

The samples were tested at various pH values to find the highest recovery. Fig. 1 shows the effect of pH on the recovery of pharmaceuticals. It was observed that pH has a small effect on the recovery for some of the compounds such as acetaminophen and caffeine. The highest recovery for simvastatin, diclofenac, and mefenamic acid was at pH 7, while the recovery for metformin, nifedipine, and salbutamol was the highest at pH 10. Generally, a basic compound such as metformin is present in a dissociated form when the pH of the aqueous solution is <p*K*_a_ of the compound, which reduces the potential for trapping the analytes from the water sample at low pH. Ibuprofen and perindopril had the highest recovery at low pH, which is normal for acidic compounds at low pH due to their existence in associated form at this pH.^7^ For this study, pH 7 was chosen because the HLB sorbent has yielded satisfactory recoveries for most of the compounds.

#### 3.2.3 Choice of eluent

**Fig. 2:**
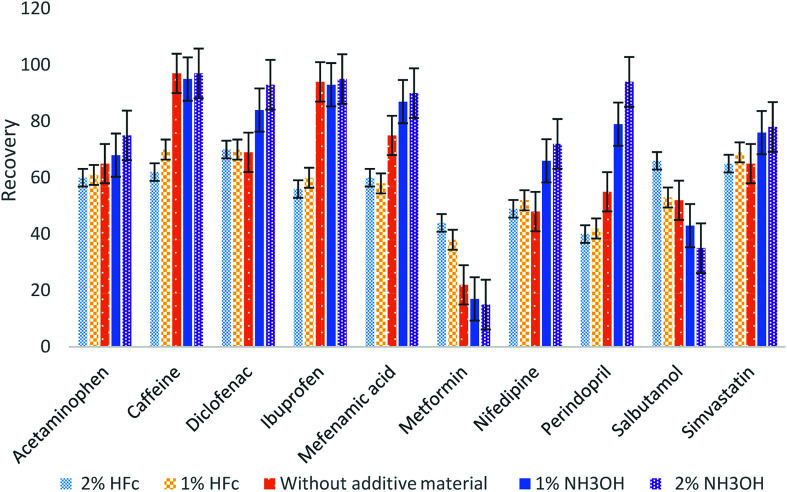
Selection of the elution additive material.

The optimum eluent material and additive materials have been chosen by testing different elute materials with several additive materials to obtain the best recovery. Our results indicated that the highest recovery values for most of the pharmaceuticals were obtained using methanol and acetonitrile mixture (1 : 1 v/v). Different materials at different concentrations were added to the eluting material (2% and 1% of formic acid, without additive material and 2% and 1% ammonium hydroxide) and were examined to determine the best additive material. Fig. 2 illustrates that the best recoveries for most of the analytes occurred at 2% ammonium hydroxide, except for metformin and salbutamol having the maximum recoveries with 2% formic acid. To ensure the optimum recovery for all pharmaceuticals, four eluting steps were used: two with 2% formic acid and then two steps with 2% ammonium hydroxide.

### 3.3 Method validation

The method was validated for tap water, influent and effluent wastewater. The calibration curves were linear in the studied range with correlation coefficients (*r*) ranging from 0.98 to 0.999 (Table S1, ESI†). The relative standard deviation (% RSD) for five replicate samples at 3 concentration levels of each matrix were calculated to evaluate the method's intra-day precision, where the range for tap water was 3–12%, and effluent and influent wastewater at 4–9% and 2–8%, respectively (Table 3). Inter-day precision was obtained by extracting and analysing one sample at 3 concentration levels of each matrix in five days and the % RSD was calculated. Inter-day precision was in the range of 6–14%, 6–14% and 6–16% for tap, effluent and influent wastewater, respectively.

**Table 3:** Performance of the developed multi-class pharmaceutical residues method

Compound	LOD (ng L^−1^)			LOQ (ng L^−1^)			Intra-day (%) RSD			Inter-day (%) RSD			Recovery (%)			*r*
	TW	EF	IN	TW	EF	IN	TW	EF	IN	TW	EF	IN	TW	EF	IN	
Acetaminophen	2.7	2.8	3.1	9.0	9.0	10.0	8	8	6	8	13	10	81	79	72	0.99
Caffeine	0.02	0.02	0.02	0.1	0.1	0.1	4	5	2	7	6	6	102	97	101	0.99
Diclofenac	0.3	0.3	0.3	0.9	1.0	0.9	5	5	6	7	9	8	118	111	121	0.99
Ibuprofen	3.2	3.0	4.7	10.9	9.9	15.7	6	8	7	9	8	6	98	107	93	0.99
Mefenamic acid	0.3	0.3	0.4	0.8	0.9	1.2	3	5	6	6	8	10	122	121	103	0.99
Metformin	0.3	0.3	0.3	0.9	0.9	0.9	9	8	8	13	14	16	48	47	47	0.99
Nifedipine	3.0	3.1	3.7	10	10.3	12.3	12	7	5	14	14	14	76	75	64	0.98
Perindopril	0.3	0.3	0.3	0.9	1.0	0.9	4	6	6	9	10	11	114	107	117	0.99
Salbutamol	0.3	0.3	0.3	1.0	1.1	1.1	6	4	7	10	9	12	70	62	62	0.99
Simvastatin	0.3	0.3	0.5	1.0	1.0	1.5	4	9	6	6	11	11	83	81	72	0.99

In this study, the absolute recovery was calculated, which was determined by comparing the peak area ratio of the analyte after extraction with those of non-extracted solutions containing the same concentration of the analyte. This is unlike most of the other studies using relative recovery, which is the percentage amount of pharmaceuticals recovered from the matrix with reference to the extracted internal standard (standard spiked into the same matrix). Absolute recovery can reveal the exact amount analyte lost during the analysis, in contrast to relative recovery which is used to compensate for the loss of the sample analyte without evaluating the real loss.^36^

The absolute recoveries were obtained by spiking three replicates of each sample matrix at two concentration levels and then the analysis method was applied. Most of the acidic compounds had an absolute recovery that was a much higher value than previously reported,^29,30,32,37–40^ even though their methods were targeting similar groups of the compound. For metformin, as expected, the recovery was low due to the high polarity of the compound (p*K*_a_ = 12.4, log *K*_ow_ = −0.5), which led to a high solubility in water and poor solubility in lipids. Thus, it is difficult to extract metformin in an aqueous matrix. However, this study is the first to report a quantitative analytical method for metformin using SPE, which is applicable for wastewaters at this level of sensitivity. For quantification analysis in real samples, an external calibration method was used to calculate the concentration of all the analytes, and since the external calibration method did not compensate for the loss of the analytes during sample preparation and chromatographic analyses, the absolute recoveries were taken into account in the quantitative calculation to compensate for the loss.

### 3.4 Matrix effect

Eliminating the matrix effects is one of the main challenges in LC/MS studies due to the negative impact on the accuracy, robustness and precision of the method. Matrix effects in LC/MS will suppress or enhance the analyte signal during electrospray ionisation due to the co-eluting matrix components in the ionisation step and the solvent additives in the mobile phase components.^41^ Suppression or enhancement of the signals can be evaluated by spiking the SPE extracts of tap water, influent and effluent wastewater using the standard mixture of pharmaceuticals, followed by LC/MS-MS, then comparing the peak areas of each compound with the peak areas of the standards.^42,43^ In this study, diluting the sample before the injection was implemented to eliminate the matrix effect.

**Fig. 3:**
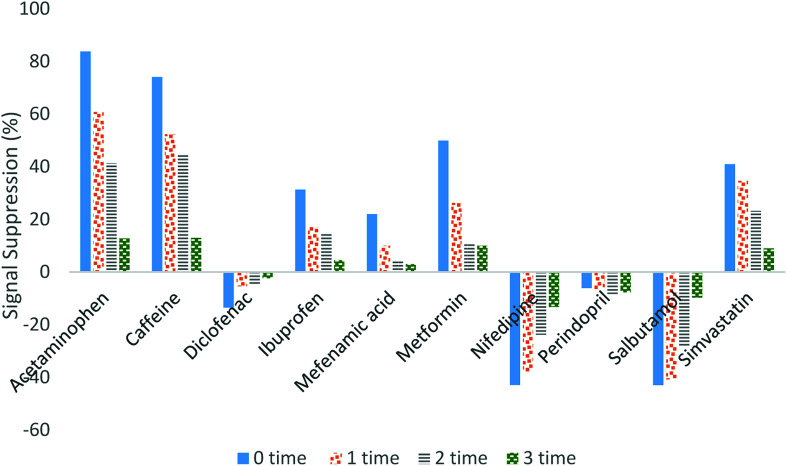
Effect of spiked SPE extract dilution on the signal suppression.

Fig. 3 illustrates how the signal suppression in wastewater was reduced when the sample was diluted. By comparing the signal suppression for undiluted and 3-times diluted samples, the efficiency of the technique in eliminating the matrix effect can be observed. Based on our research findings, the signal suppression for diclofenac, ibuprofen and mefenamic acid was reduced within the range of 13 to 2%, 31 to 1%, and 21 to 3%, respectively. For acetaminophen and caffeine, the signal suppression was reduced from approximately 80% to 20%, whereas 50% to 15% reduction was obtained for simvastatin, nifedipine, metformin and salbutamol. Only for perindopril was the signal suppression not affected by the sample dilution. However, the signal suppression for perindopril was very small (less than 5%).

### 3.5 SPE using cartridge and disk

SPE was carried out using an HLB cartridge and an HLB disk on the selected pharmaceuticals to compare the performance of both approaches. The results obtained using disk were generally better than those obtained with a cartridge in terms of recoveries, precision, analysis time, limit of detection and limit of quantification. Using disk SPE, the analysis time was reduced to less than half of the analysis time of the cartridge SPE due to the fast sample flow rates for sample loading in disk extraction as compared to the cartridge.

Better recoveries were observed using the disk as compared to the cartridge on the same adsorbing materials (HLB). The results in Table 4 show the recovery increased to more than 50% for five pharmaceuticals when the disk was used. The most notable differences in recovery were for acetaminophen, salbutamol, caffeine, metformin, and perindopril, where the values were doubled for perindopril and caffeine, 15 to 20 times for acetaminophen and salbutamol, and more than 40 times for metformin. The recoveries for diclofenac, mefenamic acid and ibuprofen by disk were close to those obtained with a cartridge in the range of 90 to 120%. The improved recovery is attributed to the disk design. Membranes eliminate the channeling and the small particle size increases the path length, which leads to an increase in the interactions between the analytes and the adsorbing material in the disk. This reduces the risk of analyte loss, which can happen with other packed particle beds, such as the cartridge.

**Table 4:** A comparison of the performance of disk SPE and cartridge SPE in tap water, and the signal suppression in influent wastewater

Compound	Recovery (%)		LOD (ng L^−1^)		LOQ (ng L^−1^)		Intra-day (%) RSD (100 ng L^−1^)		Inter-day (%) RSD (100 ng L^−1^)		Signal suppression (%)	
	Disk	Cartridge	Disk	Cartridge	Disk	Cartridge	Disk	Cartridge	Disk	Cartridge	Disk	Cartridge
Acetaminophen	81.0	3.3	2.7	30.8	9.0	102.0	8	9	8	11	8	26
Caffeine	102	54	0.02	15.9	0.1	53.0	4	3	7	7	9	−26
Diclofenac	118	105	0.3	0.4	1.0	1.1	5	4	7	5	6	−19
Ibuprofen	98	95	3.3	35.1	10.9	117.0	6	8	9	7	6	23
Mefenamic acid	122	110	0.3	0.4	0.9	1.2	3	5	6	6	5	12
Metformin	48	1	0.3	145.7	0.9	485.0	9	14	13	16	17	24
Nifedipine	76	34	3.0	0.4	10.0	1.3	12	14	14	17	22	16
Perindopril	114	65	0.3	3.4	1.0	11.4	4	9	9	11	7	−5
Salbutamol	70	4	0.3	3.1	1.0	10.0	6	8	10	11	−9	−19
Simvastatin	83	60	0.3	3.7	1.0	12.3	4	6	6	7	16	31

By comparing the precision (intra-day and inter-day) of the two methods, we can see the difference between the disk and the cartridge, where the disk was more precise than the cartridge in all the analytes except for diclofenac, mefenamic acid and ibuprofen where it was similar. The disk method produces clean matrices and a concentrated analyte solution that enhances the signal-to-noise ratios, leading to improved the limits of detection and quantitation in LC/MS/MS analysis. LOD and LOQ were lower for the disk method as compared to the cartridge method for most of the pharmaceuticals except for the diclofenac and mefenamic acid, where they were almost similar in both methods. Nifedipine was the only compound that had higher LOD and LOQ in the disk compared to the cartridge.

Our research findings revealed that the disk SPE gave better results in cleaning up and significantly reduced the matrix effect. Table 4 shows the low signal suppression observed when the disk was used for most of the pharmaceuticals when compared to the cartridge. For instance, the matrix effect for influent wastewater with the disk ranged from -9–22%, while it was -26–31% for the cartridge. This difference could be related to the small particle size that made the disk worked as a filter membrane, which produced clean extracts and minimised fine particles potentially reaching the LC/MS/MS. The only disadvantage of using disk SPE in this study is the high price of the disk as compared to the price of the cartridge. On the other hand, using the disk SPE reduced the cost of the solvent and the manpower, which can reduce the analysis cost. The results of using the disk SPE compared favorably with the literature as shown in Table 5.^27,29,30,32,37–40^

**Table 5:** Comparison of the performance of the new method in the current study and previous studies^a^

Sample type	Analyte	Analysis method and technique	IDL (pg)	Recovery	LOD (ng L^−1^)	LOQ (ng L^−1^)	Intra-day%	Inter-day%	References
Wastewater/tap water	All the analytes	HLB SPE disk LC-MS/MS (qqq)	9.7–59 (fg)	62 to 118 metformin 48	0.02–4.73	0.1–15.7	2–12	6–16	This study disk
Wastewater/tap water	All the analytes	HLB SPE cartridge LC-MS/MS (qqq)	9.7–59 (fg)	1.3–110	0.34–145.7	1.1–485	3–14	5–17	This study cartridge
Drinking and surface water	Ibuprofen	HLB SPE cartridge 200 mg 6 mL LC-MS/MS (qqq)	0.5–20	61–93	NR	0.4–15	8–17	6–40	30
	Mefenamic acid								
	Diclofenac								
	Simvastatin								
Wastewater/tap water/river mineral	Ibuprofen	Strata-X 33U	NR	85	30	NR	18	NR	29
	Diclofenac	Polymeric reversed phase (200 mg/6 mL) LC-MS/MS (qqq)		83	20		14		
Drinking and surface water	Ibuprofen	HLB SPE cartridge	NR	174	1.0	3.8	3.5	NR	37
	Diclofenac			57	5.2	17.1	42		
	Acetaminophen			139	6	20	7.3		
	Salbutamol			30	0.9	3.0	4.4		
Hospital wastewater	Ibuprofen	HLB SPE cartridge	22	111.7	31	86	1.4	11.2	38
	Diclofenac		27	113.6	30	84	2.7	13.6	
	Mefenamic acid		2	100.1	4	11	2.4	3.1	
Drinking	Ibuprofen	(Speedisk H_2_O-Philic DVB, J.T. Baker), GC-MS-SIM	NR	92.4	1.4	3.6	NR	NR	39
	Diclofenac			89.6	2.2	7.4			
	Salbutamol			105.8	0.3	0.9			
Wastewater	Diclofenac	GC-MS SPE-DEX	NR	NR	0.098	0.098	0.119	NR	32
Wastewater	Ibuprofen	Hollow fibre liquid phase microextraction, LC-MS/MS	NR	NR	16.8 μg L^−1^	55.9 μg L^−1^	NR	NR	40
	Diclofenac				7.1 μg L^−1^	23.6 μg L^−1^			
River water	Ibuprofen	OASIS HLB LC-MS/MS	NR	NR	NR	19		5.4	27
	Diclofenac					15		4.1	
	Caffeine					39		12	

a NR: not reported.

### 3.6 Analysis of environmental samples

The new analytical method was successfully applied in tap water and wastewater. Many studies have reported the occurrence of pharmaceutical residues in wastewater from conventional wastewater treatment plants. There are only a few studies on the wastewater from hospital treatment plants. In this study, influent and effluent wastewater samples were collected to determine the concentrations of the selected pharmaceuticals in order to assess the efficiency of the treatment process in the hospital treatment plant. To ensure the presence of the targeted pharmaceuticals in the wastewater, the 10 chosen pharmaceuticals in this study belong to the 40 most commonly used pharmaceuticals in Malaysia. Three sample batches were collected: the first batch was collected during the dry season (October 2016) and the other two batches were taken in the rainy season (May and Aug 2017). Table 5 shows the mean concentrations of the targeted pharmaceuticals in tap water and the three sampling seasons for the influent and effluent wastewaters and the treatment efficiency.

All of the targeted pharmaceuticals were detected in the influent and effluent samples in three sampling trips in various concentrations except for nifedipine and perindopril, which were not detected in the third sampling trip. In the influent wastewater, the acetaminophen concentration was very high (above 14 000 ng L^−1^) in all the sessions. Caffeine, simvastatin and metformin were abundant in the influent wastewater where the concentrations were between 1400 to 11 000 ng L^−1^. The mean concentrations of other pharmaceuticals were varied for each compound and for each sampling batch (Table 6). Contrary to the influent, effluent concentration was medium to low for most of the pharmaceuticals, which did not exceed 2000 ng L^−1^, indicating the efficiency of the removal process. In general, the highest influent concentration of most of the pharmaceuticals was during the second sampling session (May) which ranged from 51.4 ng L^−1^ (ibuprofen) to 49 423.7 ng L^−1^ (acetaminophen). Different concentrations of all the compounds in the different sampling sessions for the wastewater might reflect the changes in the consumption of these drugs in each month. Fig. S2–S11 in the ESI† show the TIC and MRM chromatograms of a standard mixture, blank, tap water sample, effluent wastewater (EF) sample and influent wastewater sample (IN).

**Table 6:** The concentrations of the pharmaceuticals in tap water, influent and effluent wastewater and the treatment efficiency^a^

Compounds	Tap water (ng L^−1^)	Samples 1			Samples 2			Samples 3		
		Effluent (ng L^−1^)	Influent (ng L^−1^)	Removal percentage (%)	Effluent (ng L^−1^)	Influent (ng L^−1^)	Removal percentage (%)	Effluent (ng L^−1^)	Influent (ng L^−1^)	Removal percentage (%)
Acetaminophen	ND	343 ± 2	23 736 ± 11	98	28 ± 1	49 424 ± 18	99	330 ± 2	14 359 ± 6	97
Caffeine	ND	1763 ± 8	4708 ± 15	62	1016.9 ± 0.7	7440 ± 7	86	1129 ± 9	3629 ± 11	68
Diclofenac	ND	164.9 ± 0.9	99.5 ± 0.4	−65	54.0 ± 0.6	101.0 ± 0.3	46	109.6 ± 0.6	112.4 ± 0.6	2
Ibuprofen	ND	261 ± 1	270 ± 2	3	41.8 ± 0.9	51 ± 2	18	202 ± 3	438 ± 7	53
Mefenamic acid	ND	678 ± 5	504 ± 3	−34	89.2 ± 0.9	259.2 ± 0.6	65	468 ± 6	518 ± 3	9
Metformin	ND	640 ± 3	2330 ± 6	72	1203.0 ± 0.8	7695 ± 1	84	390.4 ± 0.8	1421 ± 4	72
Nifedipine	ND	26.3 ± 0.4	30.9 ± 0.2	14	33.5 ± 0.2	445.2 ± 0.2	92	ND	ND	—
Perindopril	ND	112.3 ± 0.8	82.2 ± 0.5	−36	81.7 ± 0.5	252.1 ± 0.5	67	ND	ND	—
Salbutamol	ND	90.1 ± 0.4	110.6 ± 0.4	18	41.7 ± 0.3	71.0 ± 0.3	41	35.3 ± 0.4	45.0 ± 0.2	21
Simvastatin	ND	132 ± 5	5254 ± 12	97	220 ± 2	11 809 ± 13	98	82 ± 2	1719 ± 6	95

a ND: not detected.

Treatment efficiencies for the hospital treatment plant were different from one compound to another, and differed for each sampling session. The removal efficiency was evaluated by calculating the removal percentage during wastewater treatment using the following equation:^44^Percentage of removal = (influent − effluent)/influent × 100%).

Acetaminophen and simvastatin were removed at the rate of 99%. Moderate removal values were observed for caffeine and metformin (70%) for all the sampling seasons. The removal of other pharmaceuticals was in different percentages for each sampling season. Perindopril, diclofenac and mefenamic acid persisted in higher concentrations in effluent wastewater than the concentration measured in influent (untreated) wastewater in the first sampling session. The increased concentration levels of these compounds in the effluent compared to influent has been reported in other studies.^38,45–47^ This phenomenon could be explained by several theories such as the cleavage of these glucuronide compound conjugates during the treatment processes to release these drug-free forms. Another theory could explain this increase as being due to the formation of transformation products such as epoxy–derivatives and hydroxyls in the influent wastewater, which are not detected by the method used for the original drugs before the treatment, and later breaks down to yield the free form of the drug that can be detected.^47^ Moreover, these pharmaceuticals could adsorb to some organic matter in the influent, which would lead to a reduction in the free pharmaceuticals detected by the method. Consequently, the organic matter would break down and release the pharmaceuticals into the effluent wastewater after the treatment process.

## 4 Conclusion

The performance of disk SPE and cartridge SPE was compared to evaluate the best SPE format for a wide polarity range of pharmaceuticals. The method using disk SPE was better in terms of recovery, sensitivity, rapidness and matrix effect as compared to the cartridge method. Positive and negative ionisation modes were used simultaneously in the LC-MS/MS analysis of the targeted analytes in a single run. The disk SPE method was successfully applied for the detection of 10 compounds with a variety of physicochemical properties in tap water and hospital wastewater. The absolute recovery was above 70% (except for metformin at 47%). The method provided high selectivity and sensitivity with low detection limits and was applied to assess the removal efficiency of the targeted pharmaceutical compounds in one Malaysian hospital's wastewater treatment plant. Most of the pharmaceuticals were detected in the influent and effluent wastewaters in different concentrations. The results also showed a variation in the treatment efficiencies for the hospital treatment plant from one compound to another.

## Conflicts of interest

There are no conflicts to declare.

## Supplementary Material

RA-008-C8RA06885B-s001
