# Supplementary material for: Disk solid-phase extraction of multi-class pharmaceutical residues in tap water and hospital wastewater, prior to ultra-performance liquid chromatographic-tandem mass spectrometry (UPLC-MS/MS) analyses
Source: RSC Adv. 2018 Dec 3;8(70):40358–68. doi: 10.1039/c8ra06885b (PMC9091434; doi:10.1039/c8ra06885b)
Supplement: RA-008-C8RA06885B-s001 [file RA-008-C8RA06885B-s001.pdf]

Electronic Supplementary Material (ESI) for RSC Advances.
This journal is © The Royal Society of Chemistry 2018

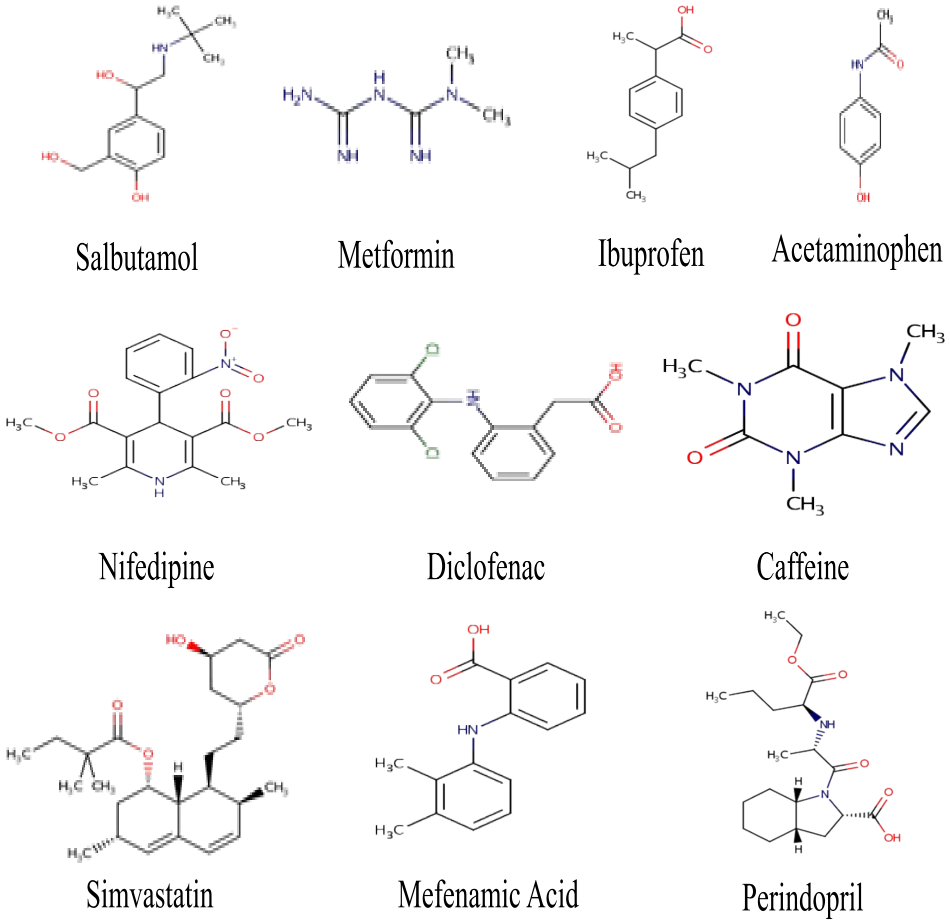

Figure S 1. Chemical structures of the selected pharmacetical

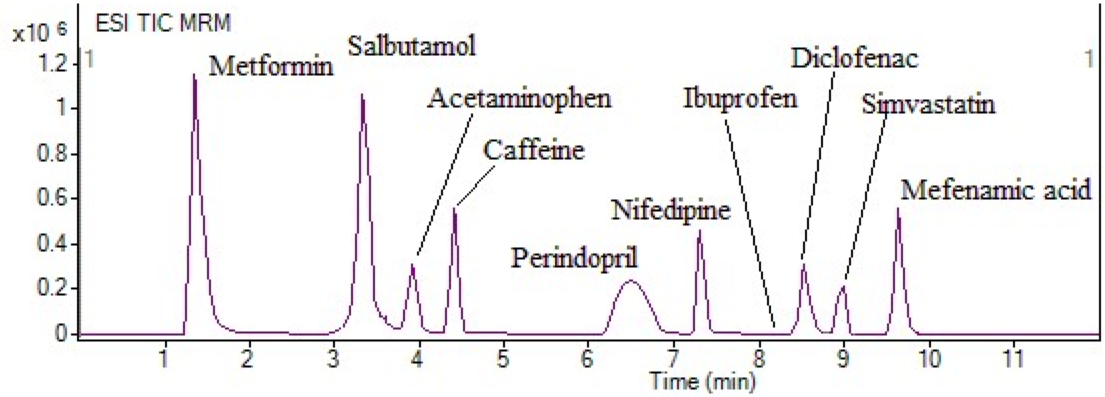

Figure S2. TIC chromatogram of a standard mixture of 1 ng μL^{-1} for targeted compounds analyzed by negative and positive ionization (ESI) modes of UPLC-MS/MS.

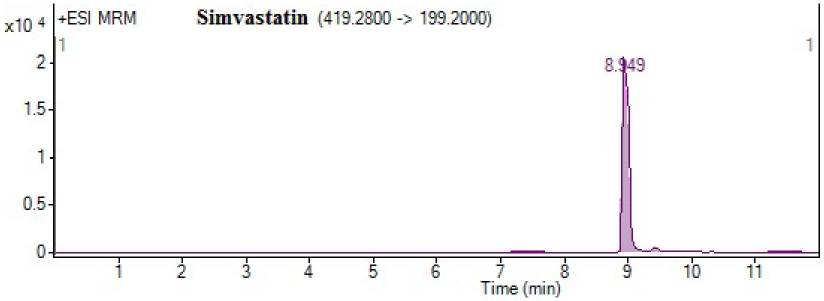

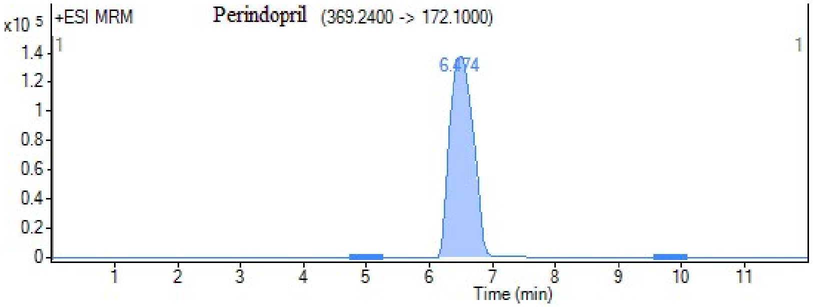

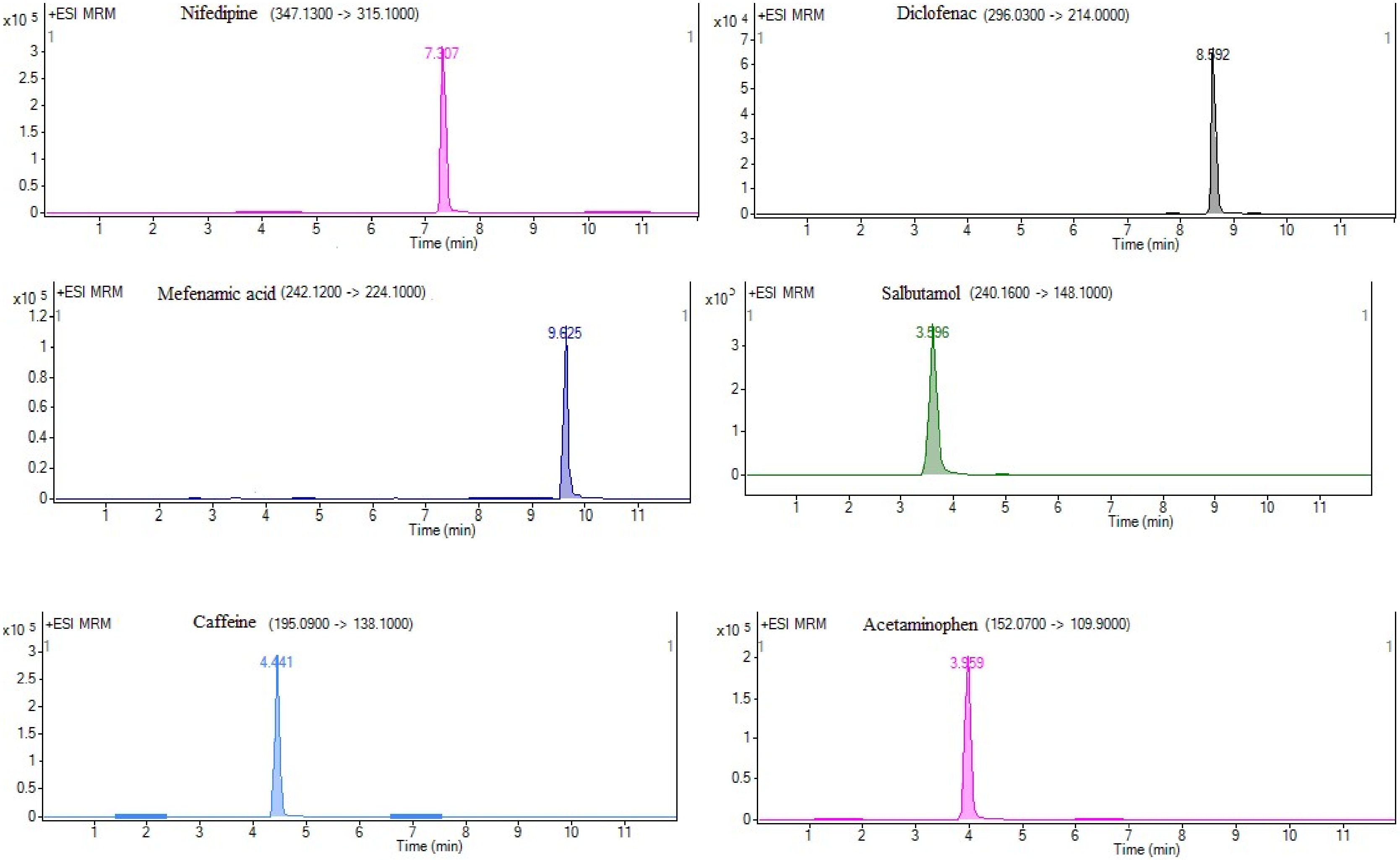
+ESI MRM
Nifedipine (347.1300 -> 315.1000)
7.307
x10 5
Time (min)
+ESI MRM
Diclofenac (296.0300 -> 214.0000)
8.592
x10 4
Time (min)
+ESI MRM
Mefenamic acid (242.1200 -> 224.1000)
9.625
x10 5
Time (min)
+ESI MRM
Salbutamol (240.1600 -> 148.1000)
3.596
Time (min)
+ESI MRM
Caffeine (195.0900 -> 138.1000)
4.441
x10 5
Time (min)
+ESI MRM
Acetaminophen (152.0700 -> 109.9000)
3.959
x10 5
Time (min)

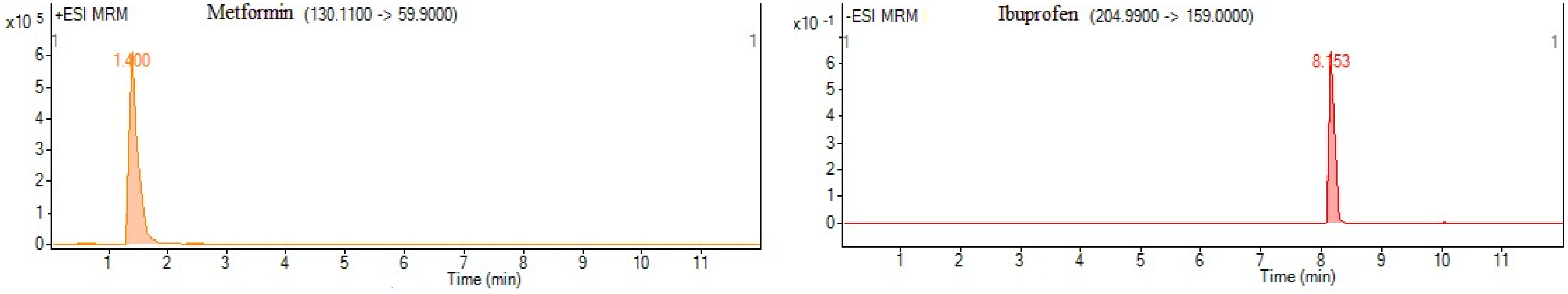

Figure S3. MRM chromatogram of a standard mixture of 1 ng μL^{-1} for targeted compounds analyzed by negative and positive ionization (ESI) modes of UPLC-MS/MS.

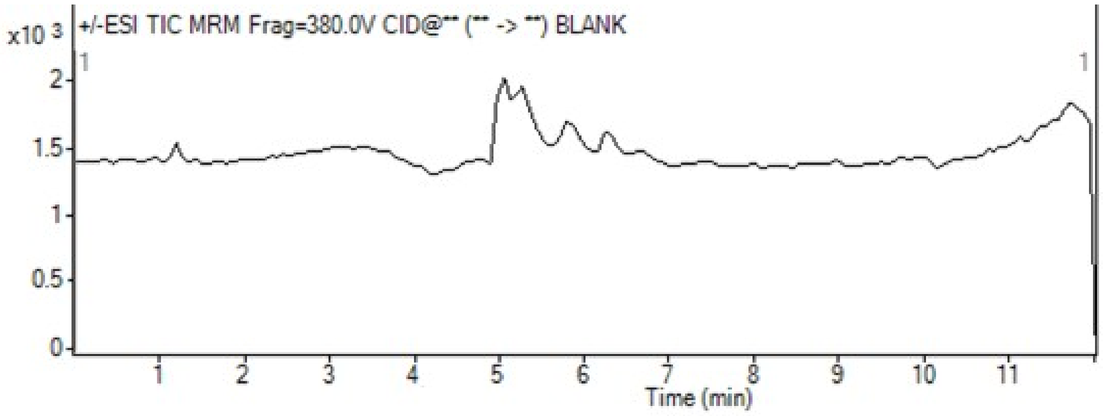

Figure S4. TIC chromatogram of a blank sample analyzed by negative and positive ionization (ESI) modes of UPLC-MS/MS.

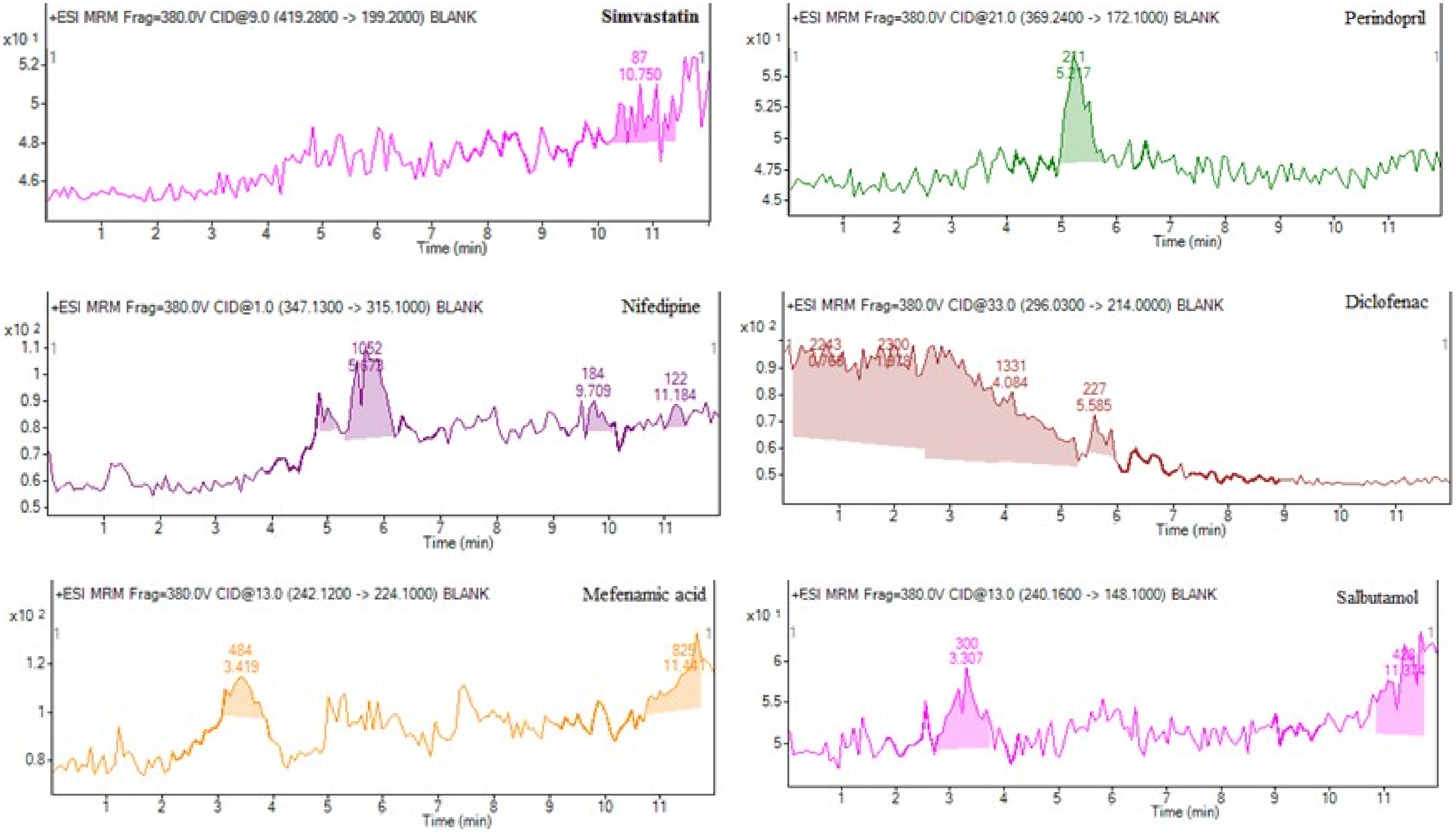
+ESI MRM Frag=380.0V CID@9.0 (419.2800 -> 199.2000) BLANK
Simvastatin
87
10.750
+ESI MRM Frag=380.0V CID@21.0 (369.2400 -> 172.1000) BLANK
Perindopril
+ESI MRM Frag=380.0V CID@1.0 (347.1300 -> 315.1000) BLANK
Nifedipine
184
9.709
122
11.184
+ESI MRM Frag=380.0V CID@33.0 (296.0300 -> 214.0000) BLANK
Diclofenac
1331
4.084
227
5.585
+ESI MRM Frag=380.0V CID@13.0 (242.1200 -> 224.1000) BLANK
Mefenamic acid
484
3.419
+ESI MRM Frag=380.0V CID@13.0 (240.1600 -> 148.1000) BLANK
Salbutamol
300
3.307
Time (min)

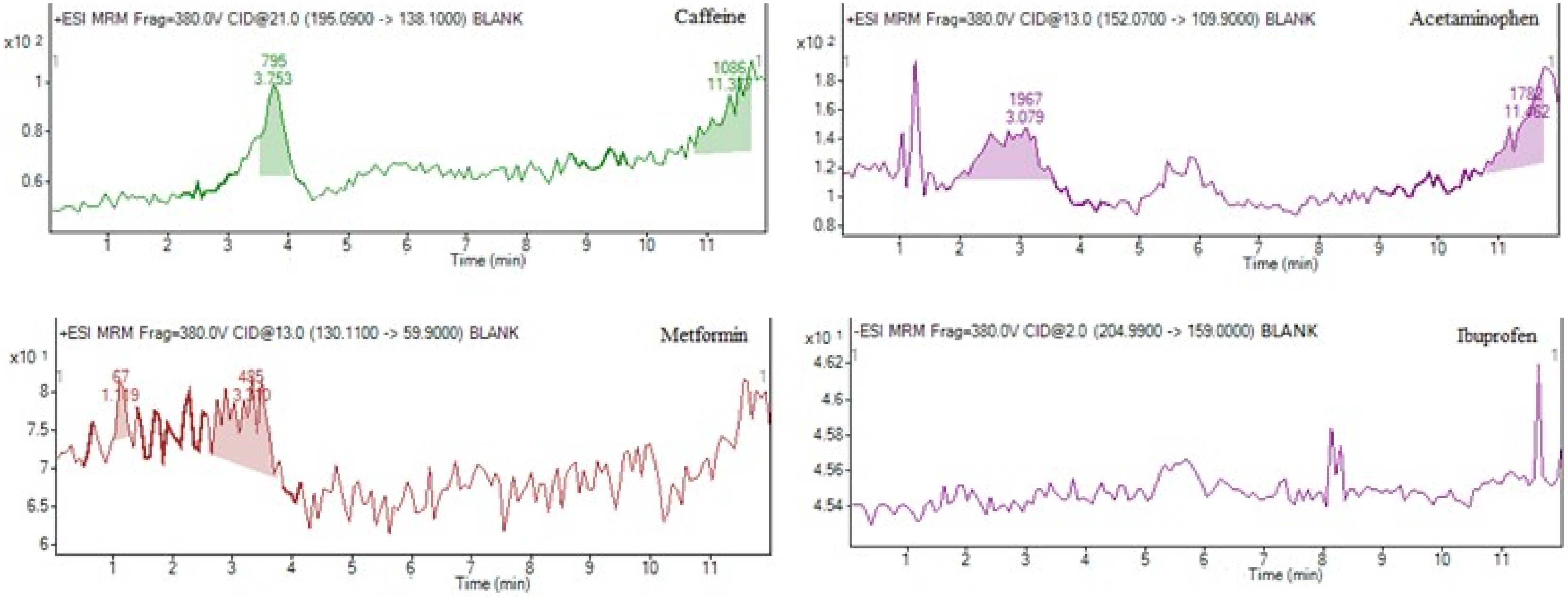

Figure S5. MRM chromatogram of a blank sample analyzed by negative and positive ionization (ESI) modes of UPLC-MS/MS.

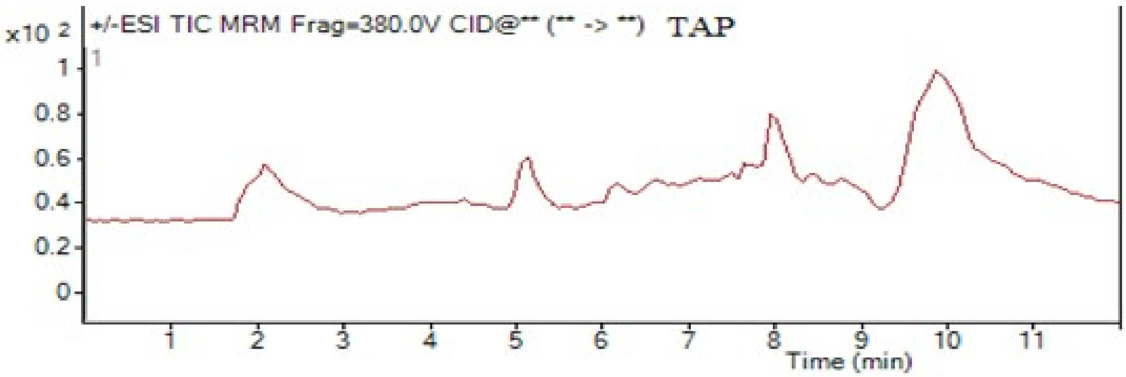

Figure S6. TIC chromatogram of a tap sample analyzed by negative and positive ionization (ESI) modes of UPLC-MS/MS.

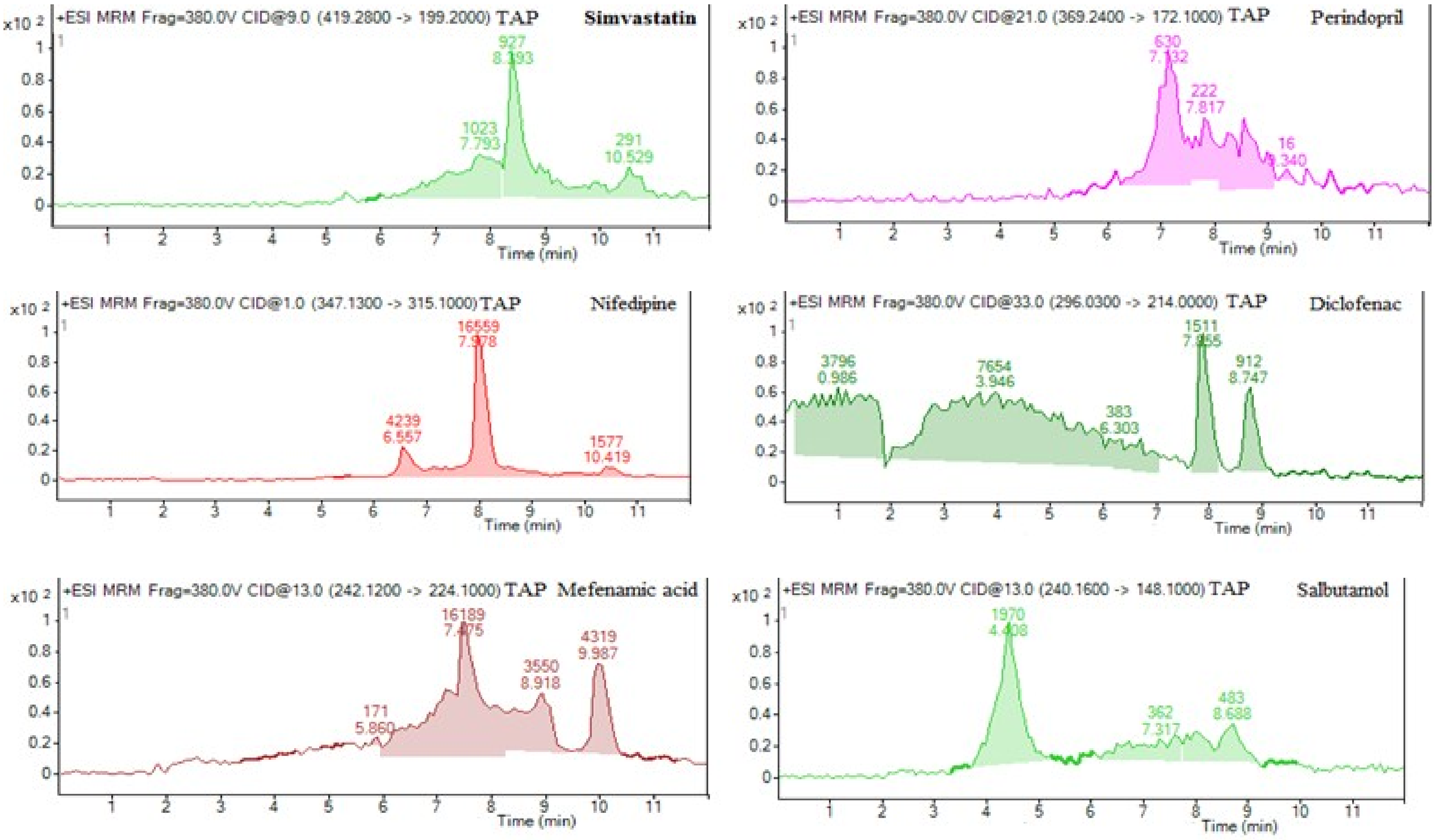

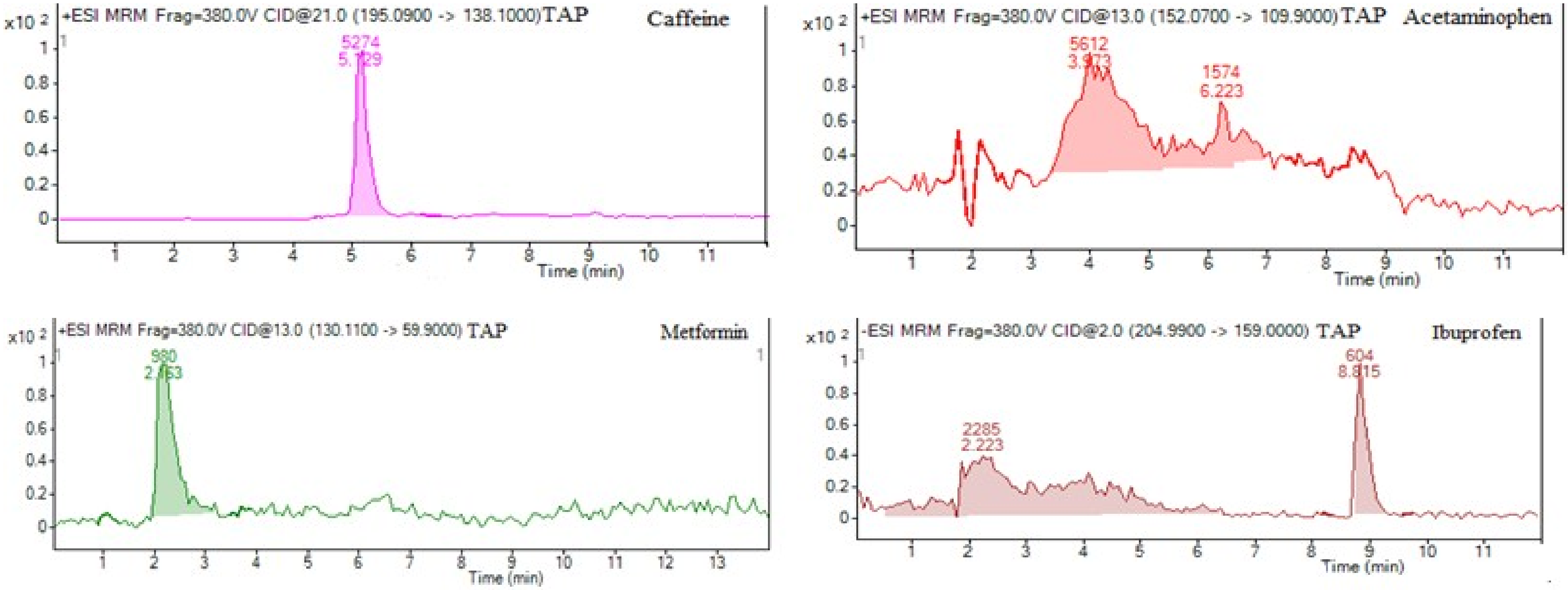

Figure S7. MRM chromatogram of a tap sample analyzed by negative and positive ionization (ESI) modes of UPLC-MS/MS.

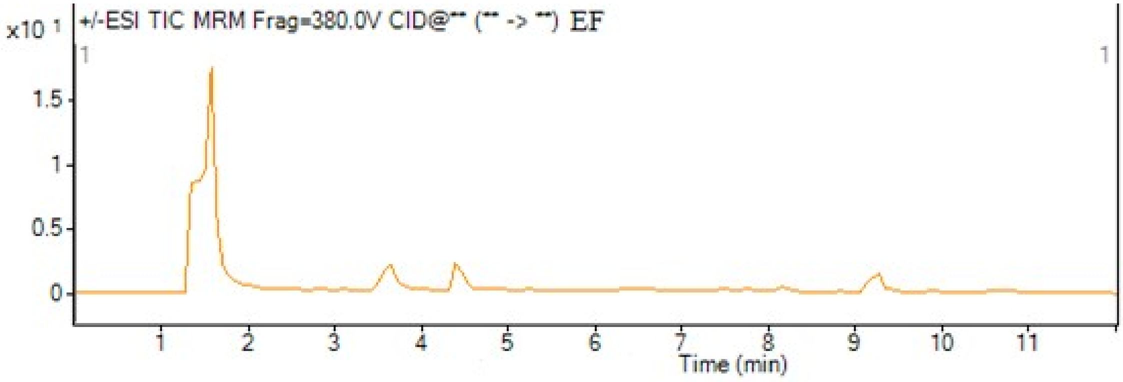

Figure S8. TIC chromatogram of an effluent wastewater (EF) sample analyzed by negative and positive ionization (ESI) modes of UPLC-MS/MS.

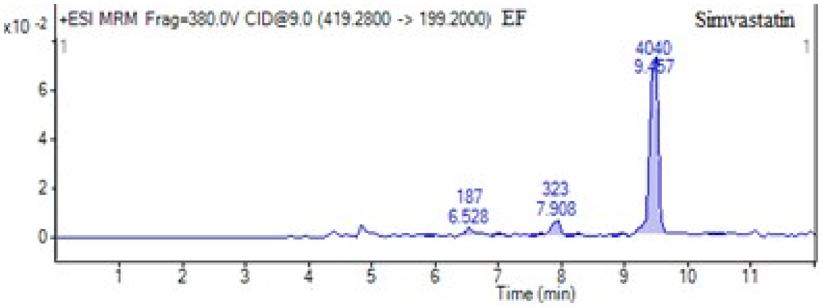

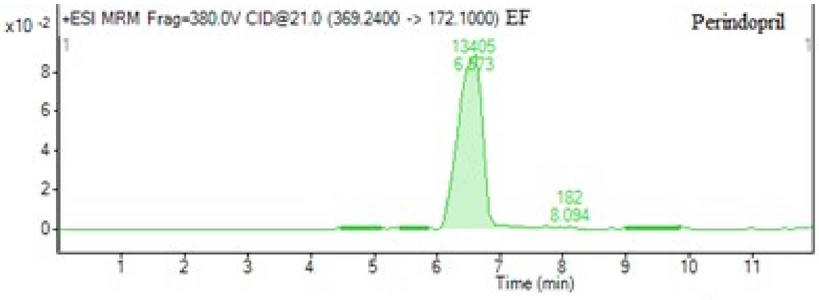

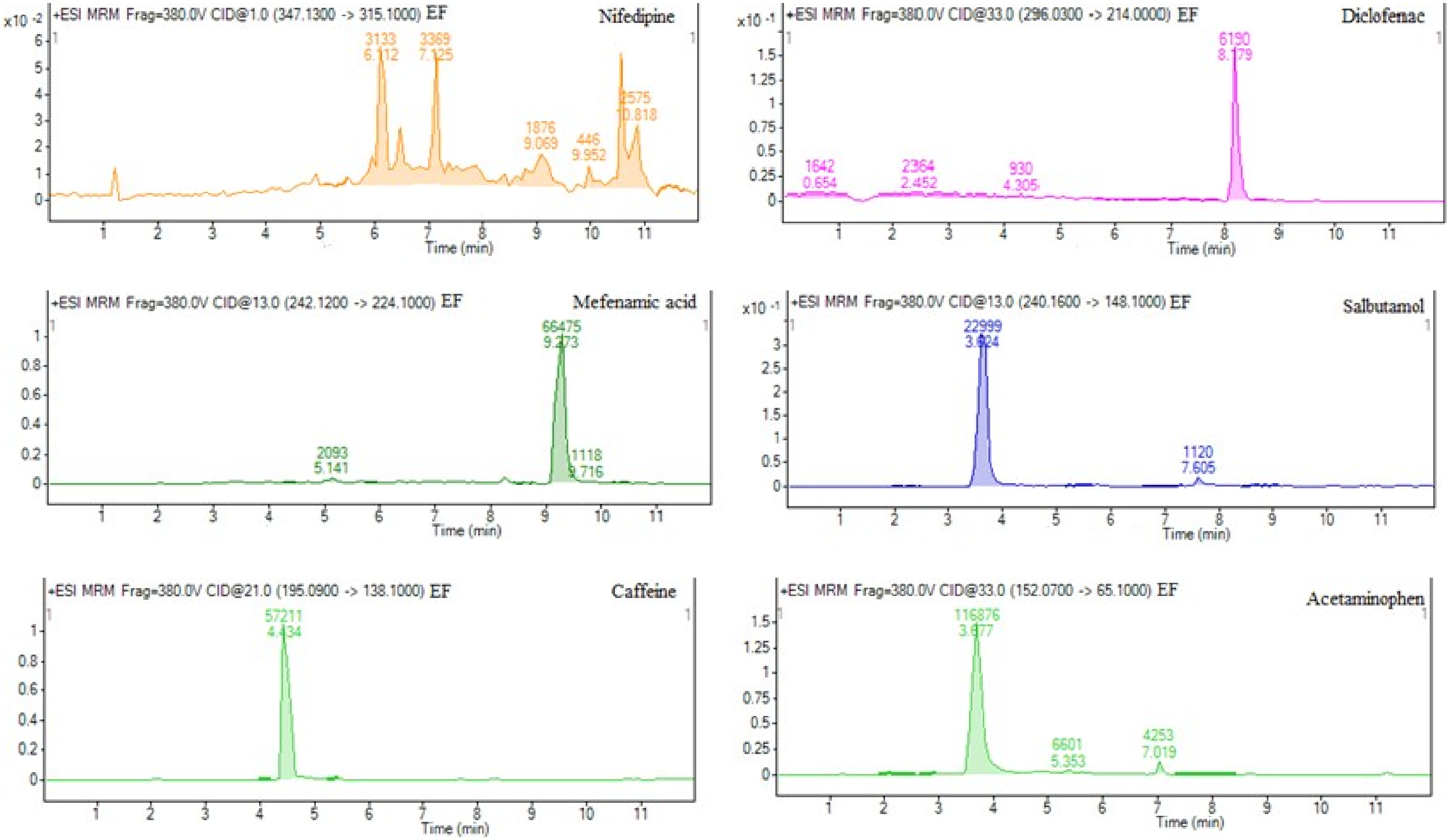
+ESI MRM Frag=380.0V CID@1.0 (347.1300 -> 315.1000) EF
Nifedipine
x10 -2
3133
6.112
3369
7.125
1876
9.069
446
9.952
2575
10.818
Time (min)
+ESI MRM Frag=380.0V CID@33.0 (296.0300 -> 214.0000) EF
Diclofenac
x10 -1
6190
8.179
1642
0.654
2364
2.452
930
4.305
Time (min)
+ESI MRM Frag=380.0V CID@13.0 (242.1200 -> 224.1000) EF
Mefenamic acid
66475
9.273
2093
5.141
1118
9.716
Time (min)
+ESI MRM Frag=380.0V CID@13.0 (240.1600 -> 148.1000) EF
Salbutamol
x10 -1
22999
3.624
1120
7.605
Time (min)
+ESI MRM Frag=380.0V CID@21.0 (195.0900 -> 138.1000) EF
Caffeine
57211
4.434
Time (min)
+ESI MRM Frag=380.0V CID@33.0 (152.0700 -> 65.1000) EF
Acetaminophen
116876
3.677
6601
5.353
4253
7.019
Time (min)

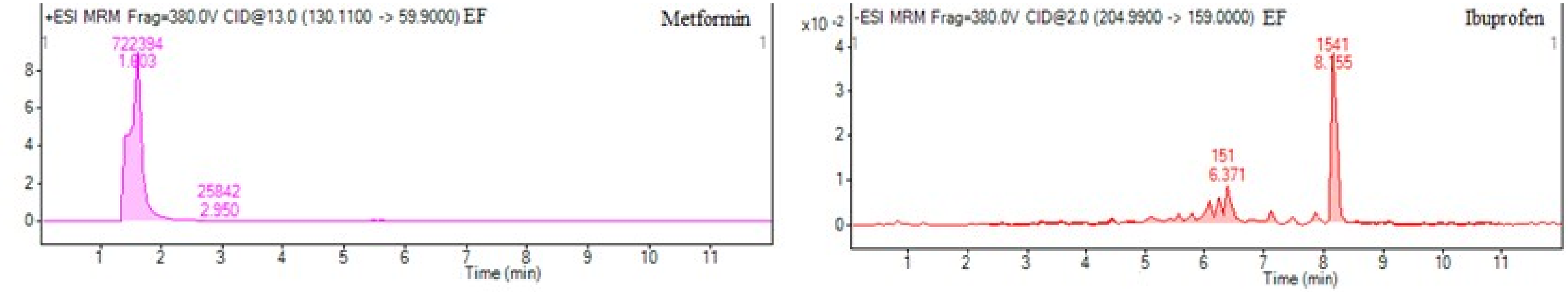

Figure S9. MRM chromatogram of an effluent wastewater sample analyzed by negative and positive ionization (ESI) modes of UPLC-MS/MS.

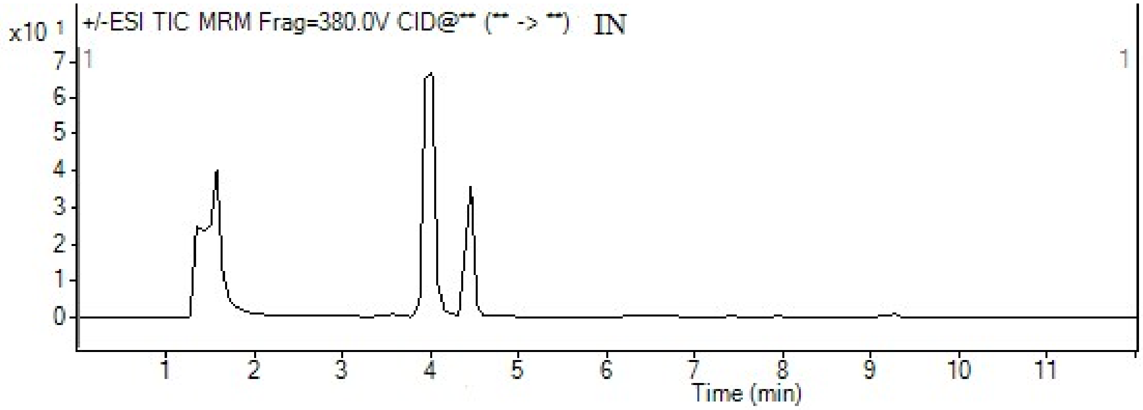

Figure S10. TIC chromatogram of an influent wastewater sample (IN) analyzed by negative and positive ionization (ESI) modes of UPLC-MS/MS.

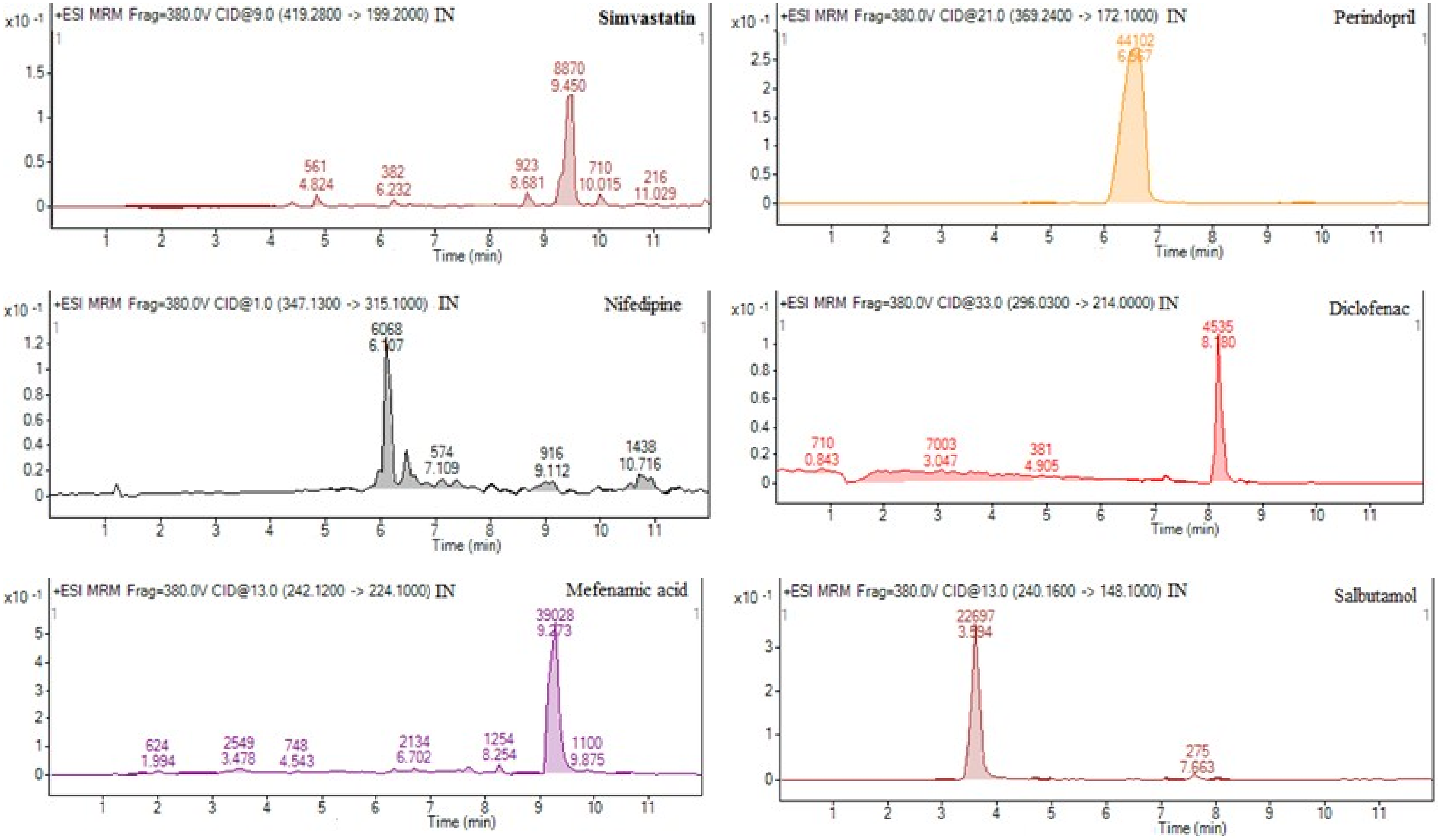

+ESI MRM Frag=380.0V CID@9.0 (419.2800 -> 199.2000) IN
Simvastatin
8870
9.450
561
4.824
382
6.232
923
8.681
710
10.015
216
11.029
+ESI MRM Frag=380.0V CID@21.0 (369.2400 -> 172.1000) IN
Perindopril
44102
+ESI MRM Frag=380.0V CID@1.0 (347.1300 -> 315.1000) IN
Nifedipine
6068
574
7.109
916
9.112
1438
10.716
+ESI MRM Frag=380.0V CID@33.0 (296.0300 -> 214.0000) IN
Diclofenac
4535
710
0.843
7003
3.047
381
4.905
+ESI MRM Frag=380.0V CID@13.0 (242.1200 -> 224.1000) IN
Mefenamic acid
39028
624
1.994
2549
3.478
748
4.543
2134
6.702
1254
8.254
1100
9.875
+ESI MRM Frag=380.0V CID@13.0 (240.1600 -> 148.1000) IN
Salbutamol
22697
275
7.663
x10 -1
Time (min)

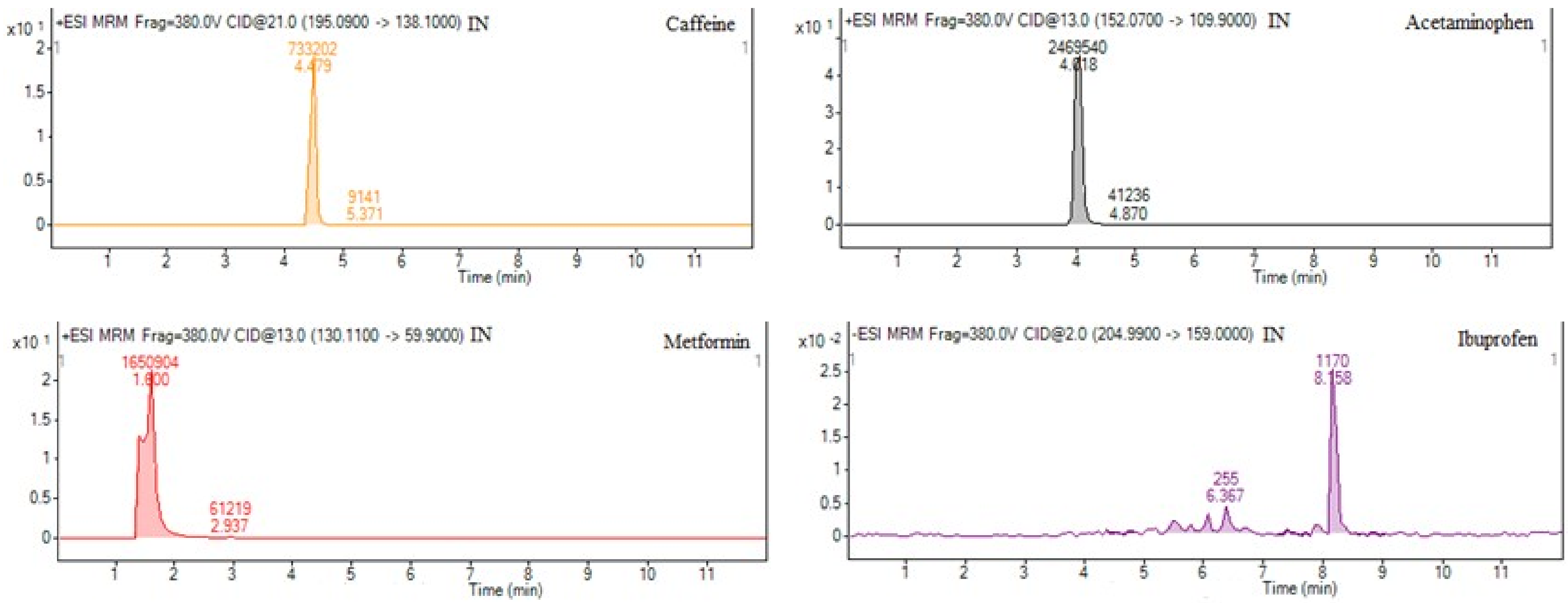

Figure S11. MRM chromatogram of an influent wastewater sample analyzed by negative and positive ionization (ESI) modes of UPLC-MS/MS.

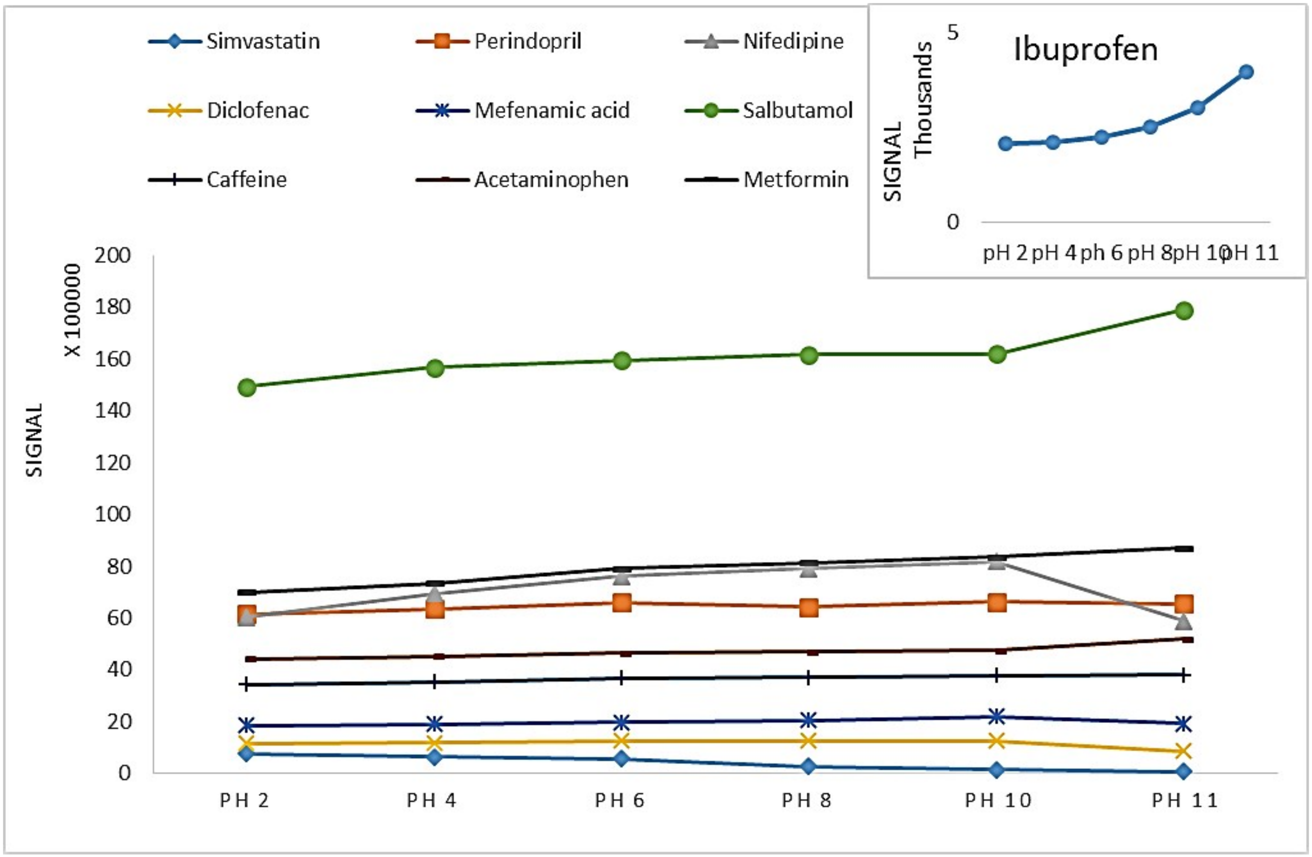

Figure S12. The effect of the matrix pH on the ionization efficiency.

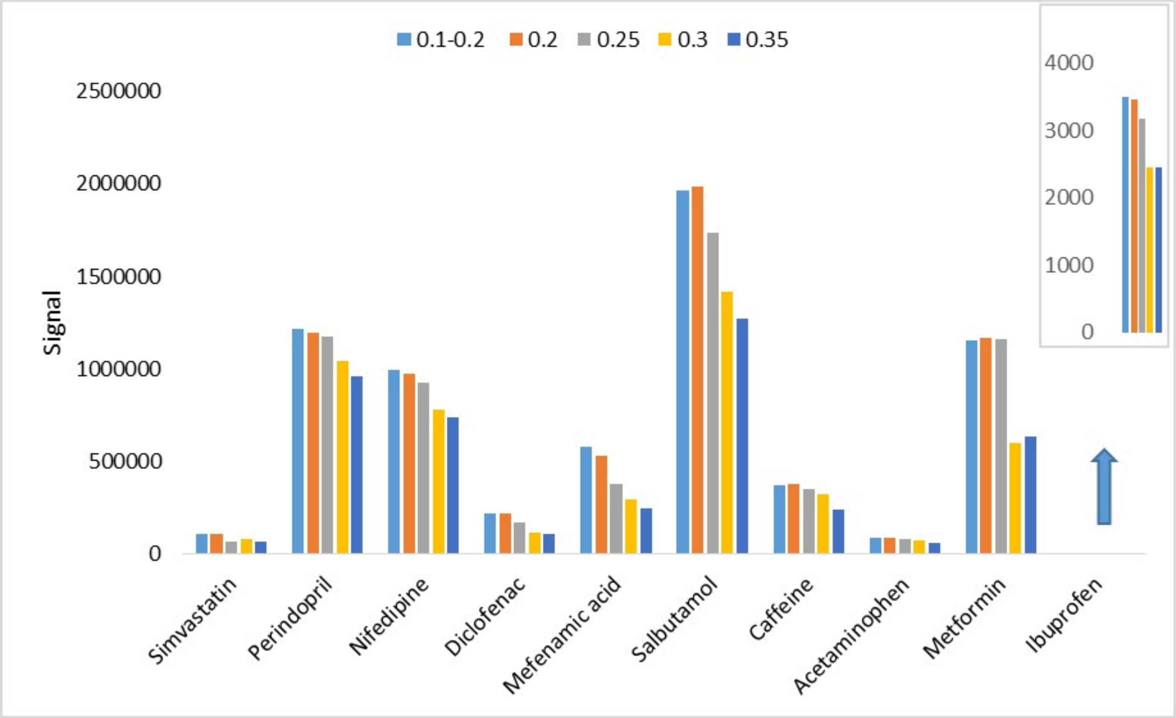

Figure S13. The effect of mobile phase flow rate on the ionization and analytes signal.

Table S1

The calibration curves details for each standard compound.

compounds	r	range μg L^{-1}	equation
Acetaminophen	0.99	0.01- 60	y = 23331x + 3478
Caffeine	0.99	0.0001-12	y = 192163x + 13991
Diclofenac	0.99	0.001-12	y = 311605x + 16861
Ibuprofen	0.99	0.01-12	y = 12664x + 592.56
Mefenamic acid	0.99	0.001-12	y = 905263x + 42920
Metformin	0.99	0.001-12	y = 283069x + 19278
Nifedipine	0.98	0.01-12	y = 466897x - 78955
Perindopril	0.99	0.001-12	y = 625466x + 16114
Salbutamol	0.99	0.001-12	y = 3E+06x + 255434
Simvastatin	0.99	0.001-12	y = 104688x + 6366.9
